# Transient sick sinus syndrome with complete atrioventricular block associated with ergonovine intake: A case report

**DOI:** 10.1097/MD.0000000000008559

**Published:** 2017-11-03

**Authors:** Hui-Ting Wang, Wen-Hao Liu, Yung-Lung Chen

**Affiliations:** a Emergency Department; b Division of Cardiology, Department of Internal Medicine, Kaohsiung Chang Gung Memorial Hospital and Chang Gung University College of Medicine, Kaohsiung, Taiwan.

**Keywords:** complete atrioventricular block, ergonovine, sick sinus syndrome

## Abstract

**Rationale:**

More mature or older women are more likely to undergo in vitro fertilization and embryo implant. These women have a greater chance of receiving ergonovine therapy because of a suspected abortion. We present this case report to call attention to a latent lethal adverse effect in everyday obstetric practice using ergonovine. It requires more attention and close monitoring

**Patient concerns:**

We presented the case of a 38-year-old female patient with general weakness and mild chest tightness after ergonovine use.

**Diagnoses:**

She was diagnosed as transient sick sinus syndrome and complete atrioventricular block with junctional escape rhythm after diagnostic work up.

**Interventions:**

Conservative treatment with discontinuation of ergonovine and bed rest.

**Outcomes:**

Her sinus rhythm returned to normal the day after ergonovine was discontinued. The patient remained symptom-free since recovery of her sinus rhythm.

**Lessons:**

Ergonovine may cause symptomatic and lethal bradyarrhythmia. Withdrawal of the causative medication and adequate supportive care can lead to a favorable outcome in these patients. More related cases should be reported. Further evaluation for treatment and prognosis are necessary.

## 1 Introduction

Ergonovine is one of the most extensively used medications in gynecology and obstetrics,^[1]^ with the effect of rapid and sustained contraction of the pregnant and non-pregnant uterus.^[2]^ It was also approved for use in diagnostic testing for Prinzmetal angina or vasospastic angina.^[3–6]^ We present the case of a patient with transient sick sinus syndrome and complete atrioventricular (AV) block with junctional escape rhythm after ergonovine use. This case report was approved by the institutional review board of Chang Gung Medical Foundation.

## 2 Case presentation

**Figure 1:**
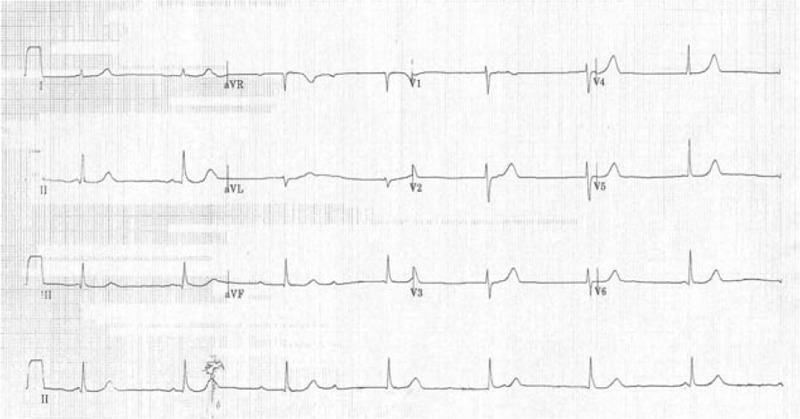
Electrocardiogram (ECG) performed at the onset of symptoms revealed complete AV block presenting with type 1 and type 2 second-degree sino-atrial exit block and junctional escape rhythm.

A 38-year-old female patient received in vitro fertilization (IVF) 1 month prior to hospitalization. Apart from primary infertility, she had no medical history of syncope, bradycardia, chest tightness, or palpitation, and her family had no history of heart disease. Swelling in the right adnexa without intrauterine embryo was found in a routine follow-up transvaginal ultrasonography. The right adnexa mass was found to be a hydrosalpinx, rather than an ectopic embryo, using exploratory laparoscopy. Vaginal bleeding presented after the laparoscopic surgery, so oral ergonovine (0.2 mg, t.i.d.) was given to facilitate uterine contraction, under the impression of complete abortion. However, by the time the patient took the fourth dose of ergonovine, she began to suffer from general weakness and mild chest tightness. The patient's vital signs were stable: systolic/diastolic blood pressure, 114/66 mm Hg; heart rate, 41 beats/min; respiratory rate, 20 breaths/min; and temperature, 36.5°C. But an electrocardiogram (ECG) showed a complete AV block presenting with type 1 and type 2 second-degree sino-atrial exit block and junctional escape rhythm (Fig. 1). The hemogram, biochemistry (including electrolytes, a series of cardiac enzyme tests, blood gas analysis, and prothrombin time), and autoimmune markers (including C3, C4, double-strand DNA, rheumatic factor, anti-beta2 glycoprotein IgG, anti-cardiolipin IgG, and IgM) were all within normal limits.

**Figure 2:**
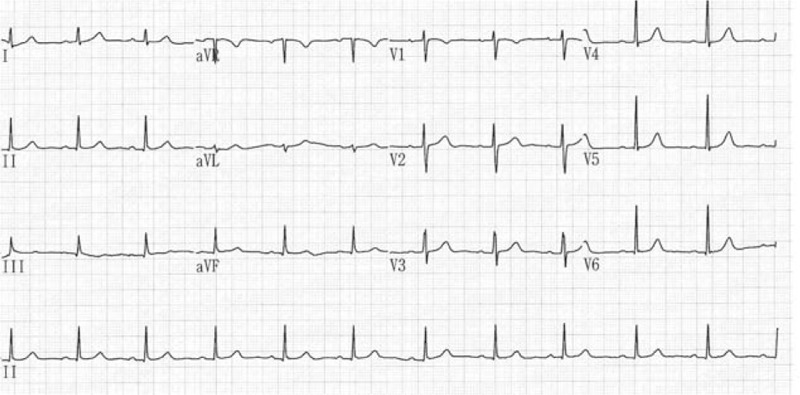
Sinus rhythm recovered the day after cessation of ergonovine.

Ergonovine was suspected to be the cause of these adverse effects since the symptoms first emerged. The Naranjo adverse drug reaction (ADR) causality score was 5, which represented probable ADR of ergonovine in this case. Conservative treatment and bed rest were suggested to the patient, and her sinus rhythm returned to normal the day after ergonovine was discontinued. ECG was arranged in a time sequence manner (Fig. 2). The patient refused further electrophysiologic study because of the IVF program. She has remained symptom-free since recovery of her sinus rhythm.

## 3 Discussion

Ergonovine currently is believed to be a useful agent that provokes coronary spasm as seen in stress ECG or angiography.^[3,7]^ The half-life of oral ergonovine is 120 minutes. There was a 25% incidence of serious ventricular arrhythmias in 95 patients receiving ergonovine for the diagnosis of variant angina.^[8]^ Ventricular fibrillation has also been reported to be caused by ergonovine-induced ischemia, which responded rapidly to cardioversion and intravenous administration of nitroglycerin.^[8]^

Common adverse reactions to ergonovine include hypertension, nausea and vomiting, dizziness, and headache. Coronary spasm and subsequent myocardial infarction in patients administered ergonovine through oral, intravenous, or intramuscular routes have been described in case reports.^[9]^ However, S-T segmental elevated myocardial infarction and Takotsubo cardiomyopathy (transient left ventricular ballooning occurring during the early postpartum period after rapid ergonovine injection) are infrequently seen.^[9–12]^ There was also a case report of postpartum severe sinus bradycardia following methylergonovine administration.^[13]^ To the best of our knowledge, this might be the first case in which ergonovine use led to transient sinus node dysfunction and complete AV block.

The mechanism of action of ergonovine is still not well understood. It may act via a calcium channel or an α-receptor in the inner myometrial layer.^[1]^ It is also a partial agonist at α adrenergic, 5HT-1, and dopamine receptors. In our case report, ECG and cardiac enzyme studies revealed no evidence of ischemic heart disease. As such, the complete AV block was not affirmatively attributed to a coronary spasm-related ischemic AV nodal branch. However, this could still be one of the possible mechanisms. Furthermore, both sympathetic and parasympathetic nervous systems contribute to the innervation of the conduction system of the heart. Vagal stimulation depresses the automaticity of the sinus node and alleviates conduction across the AV node.

Disturbances to the autonomic nervous system can explain intermittent bradycardia alone or in conjunction with an intrinsic cardiac abnormality of the sinus node or AV conduction.^[14]^ Enhanced parasympathetic tone and reduced sympathetic drive may cause sinus bradyarrhythmias, transient or permanent AV block, and neurocardiogenic syncope.^[15,16]^ Instead of direct binding to the surface membrane receptor, the ergot alkaloids act through central sympatholytic effects, resulting in bradycardia. This might provide a reasonable explanation for the resulting transient sinus node dysfunction and complete AV block in our patient. Other reversible etiologies of bradycardia include adverse drug effects, acute myocardial infarction, intoxication and electrolyte disorders.^[17]^ Adverse drug effects are the most common reversible etiology of bradycardia.^[17]^ Permanent pacemaker implantation must not be considered in patients with possibly reversible causes of bradycardia (class III indication).^[18]^ Interruption of the causative agent and waiting for a reasonable duration is the most commonly used approach for these patients. If the block is not resolved by the end of these managements, vagal ganglia ablation may be used. The vagal efferent postganglionic neurons innervating the sinus node or AV node are primarily located adjacent to the heart, and radiofrequency ablation is thought to inflict permanent damage to these neurons, but sympathetic cells are remote from the heart.^[15,16,19]^

We present this case report to call attention to a latent lethal adverse effect in everyday obstetric practice using the uterine contractant, ergonovine. More mature or older women are more likely to undergo IVF and embryo implant. These women have a greater chance of receiving ergonovine therapy because of a suspected abortion. Serious delayed side effects, including bradycardia or coronary spasm, even if atypical or in the absence of cardiovascular risk factors, require more attention and close monitoring.^[9,13]^ The effect of ergonovine might be reversed without specific intervention. Withdrawal of the causative medication and adequate supportive care can lead to a favorable outcome in these patients.^[20]^ In certain critical cases, extracorporeal membrane oxygenation can be helpful when conventional treatments are not effective.^[21]^
